# Supplementary material for: Immobilization of Metronidazole on Mesoporous Silica Materials
Source: Pharmaceutics. 2022 Oct 29;14(11):2332. doi: 10.3390/pharmaceutics14112332 (PMC9699156; doi:10.3390/pharmaceutics14112332)
Supplement: Supplementary file 1 [file pharmaceutics-14-02332-s001.zip › pharmaceutics-1933233-supplementary.pdf]

pharmaceutics

Supplementary Materials: Immobilization of Metronidazole on Mesoporous Silica Materials

Klára Szentmihályi, Szilvia Klébert, Zoltán May, Eszter Bódis, Miklós Mohai, László Trif, Tivadar Feczkó and Zoltán Károly

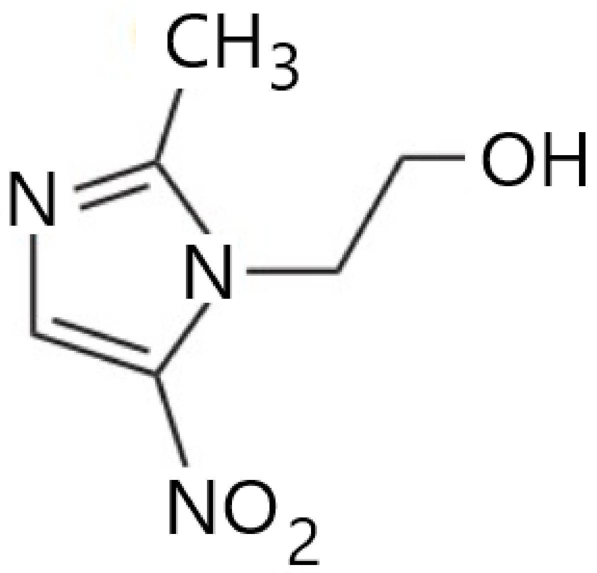

Figure S1. Structure of metronidazole.

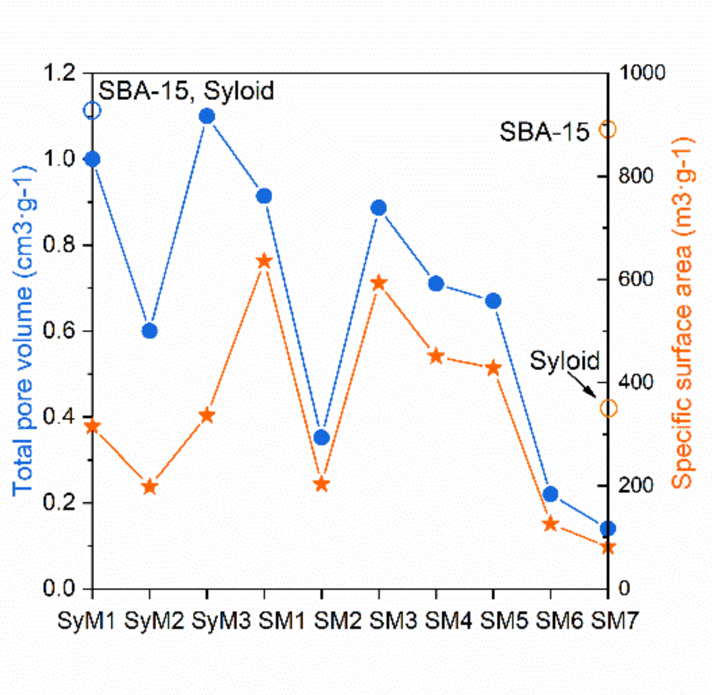

Figure S2. Total pore volume and specific surface area of the samples.

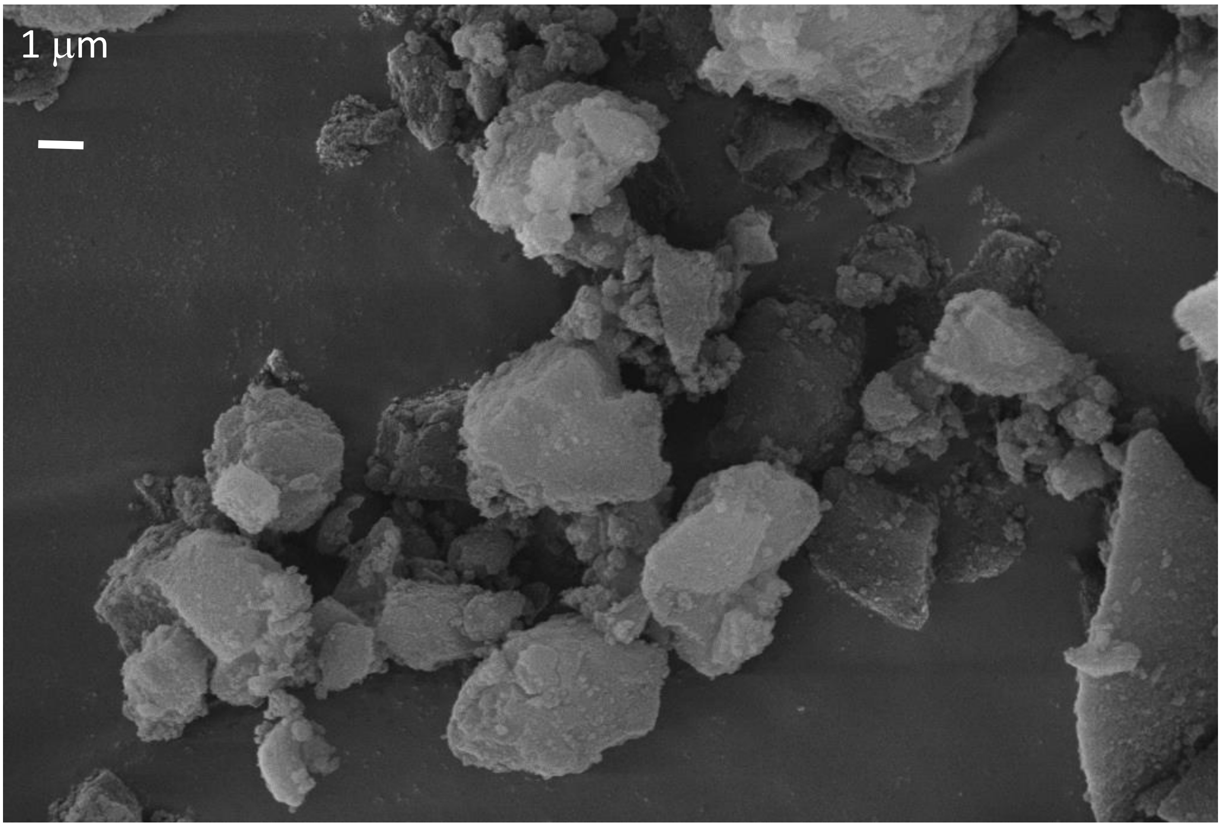

Syloid

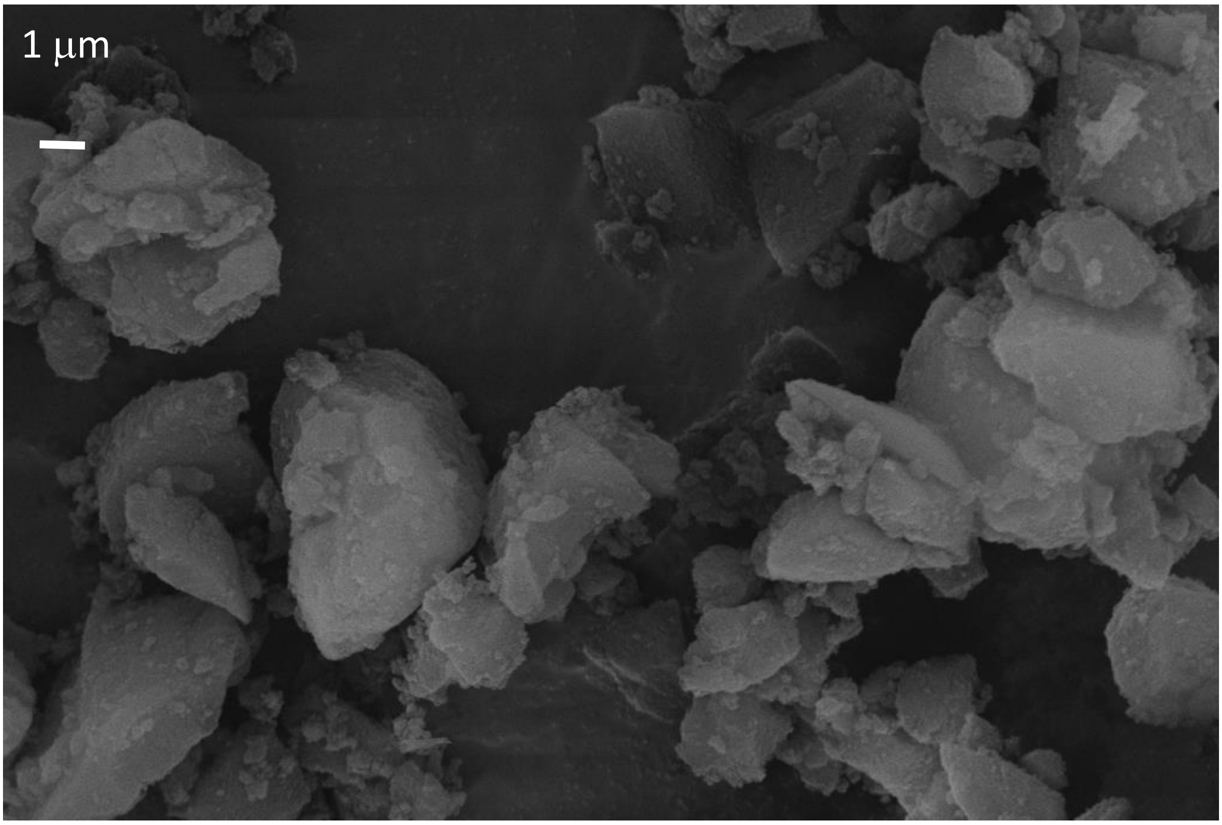

SyM1

Figure S3a Scanning electron micrographs of the Syloid and SBA-15 carriers and metronidazole immobilized on their surface**.**

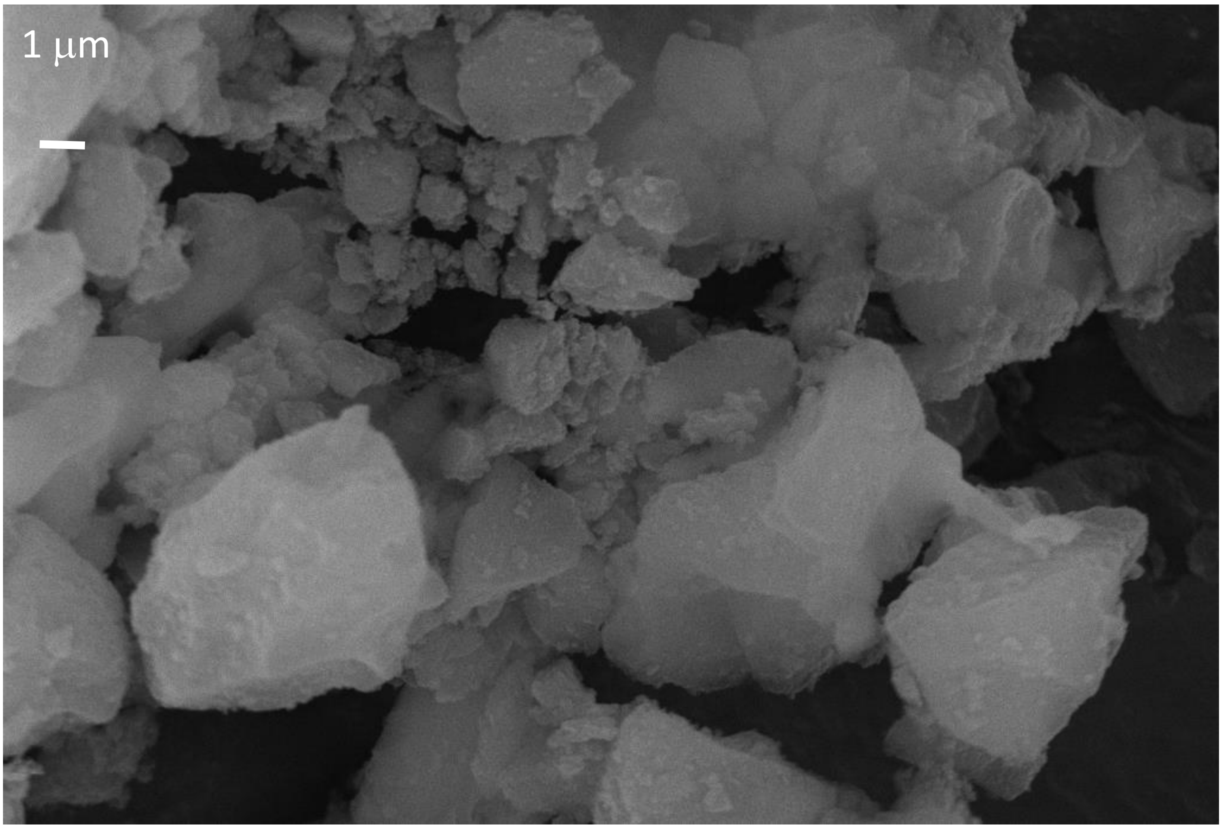

Sym2

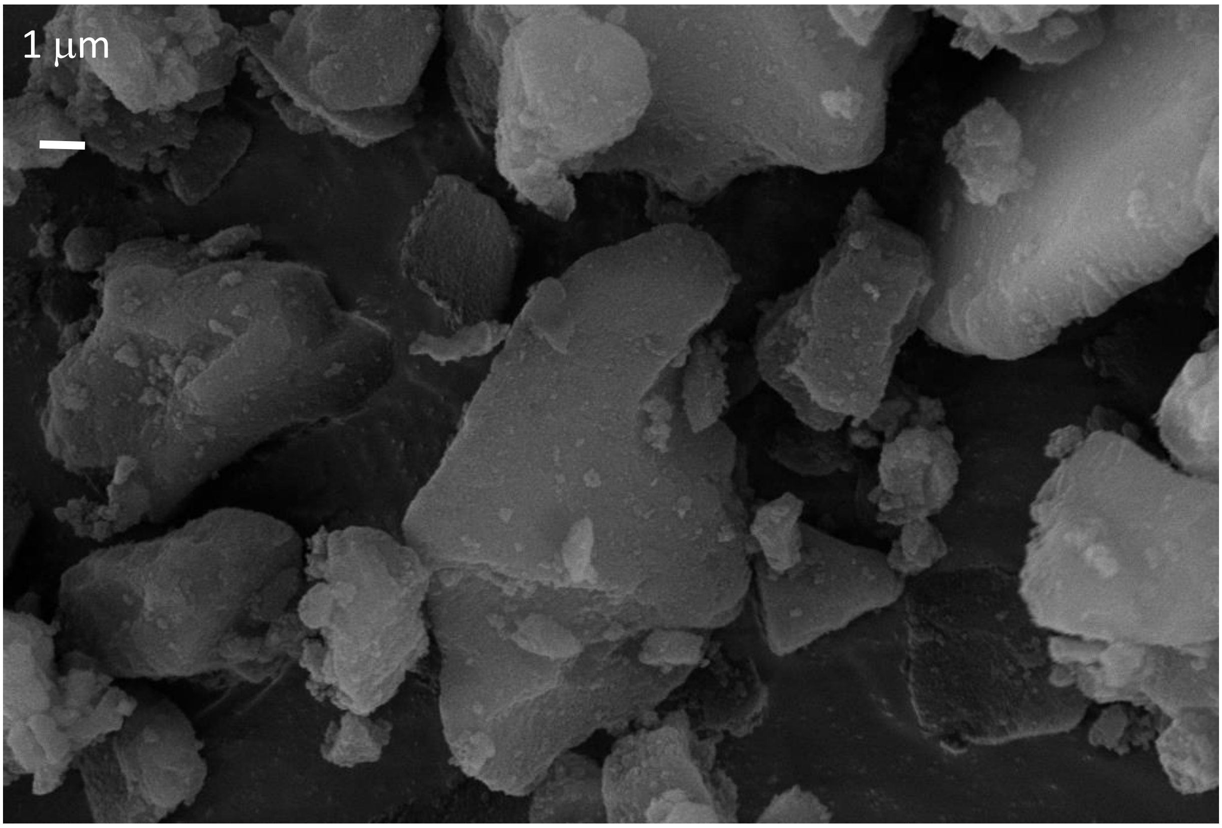

Sym3

Figure S3b Scanning electron micrographs of the Syloid and SBA-15 carriers and metronidazole immobilized on their surface.

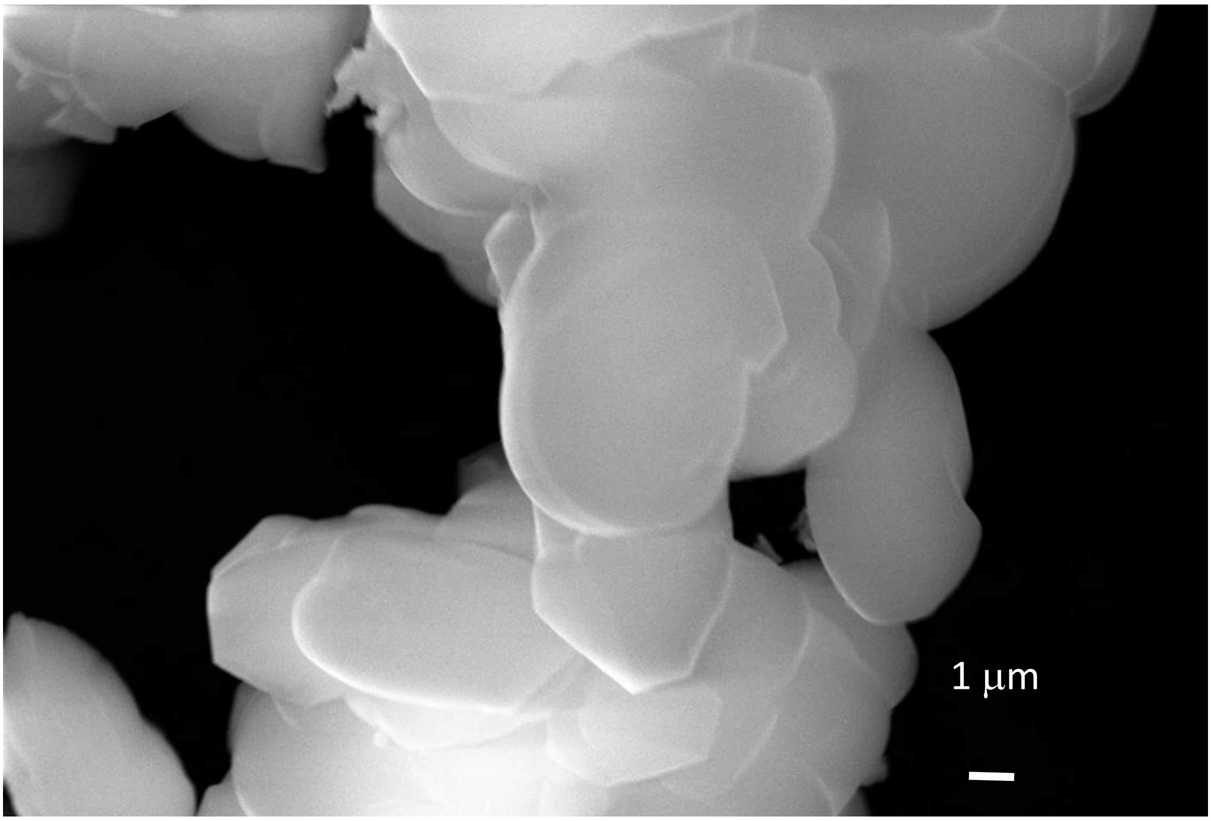

SBA-15

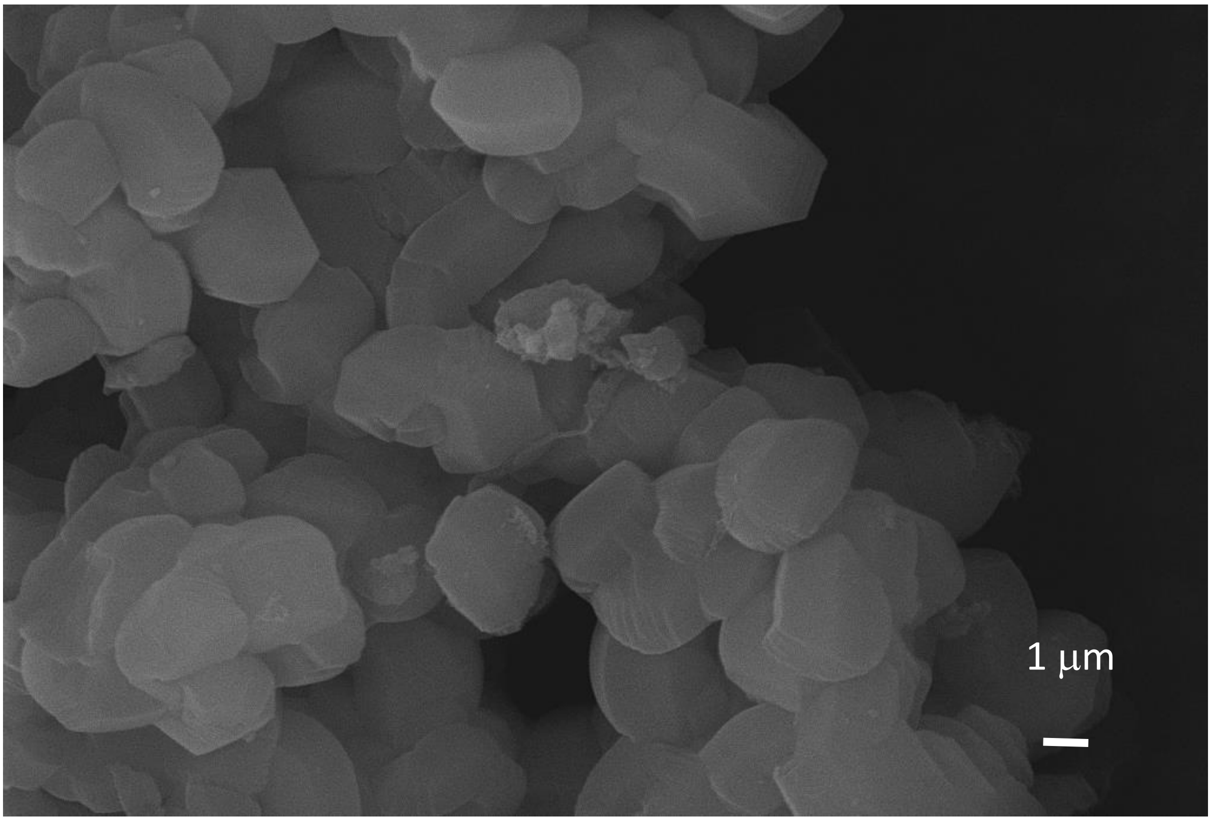

SM1

Figure S3c Scanning electron micrographs of the Syloid and SBA-15 carriers and metronidazole immobilized on their surface**.**

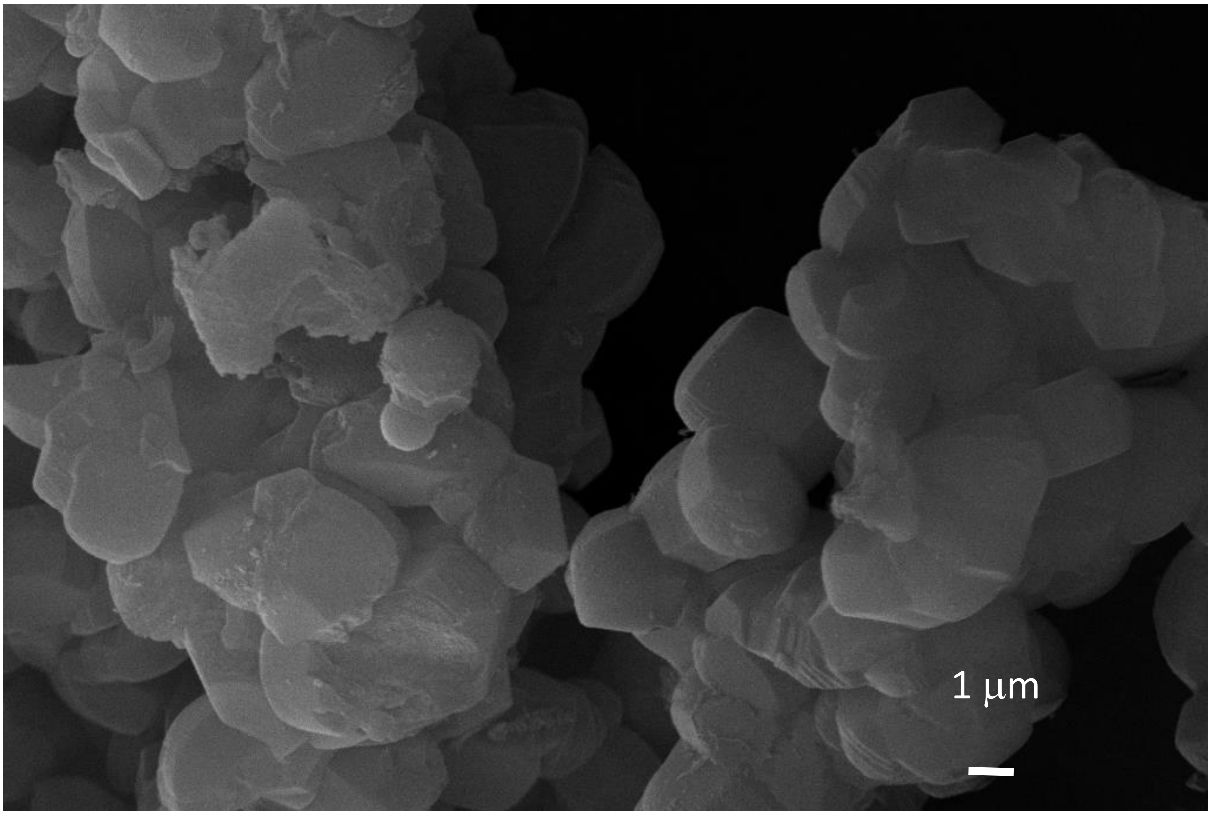

SM2

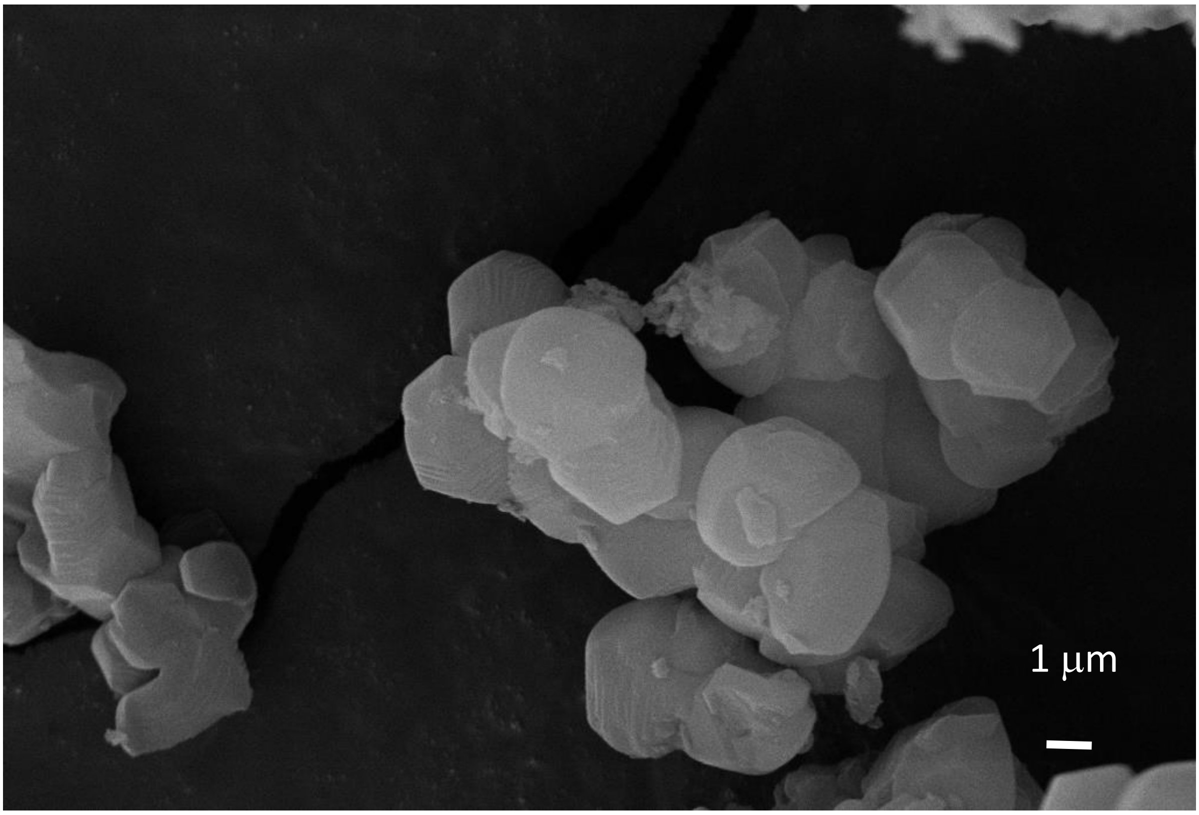

SM3

Figure S3d Scanning electron micrographs of the Syloid and SBA-15 carriers and metronidazole immobilized on their surface.

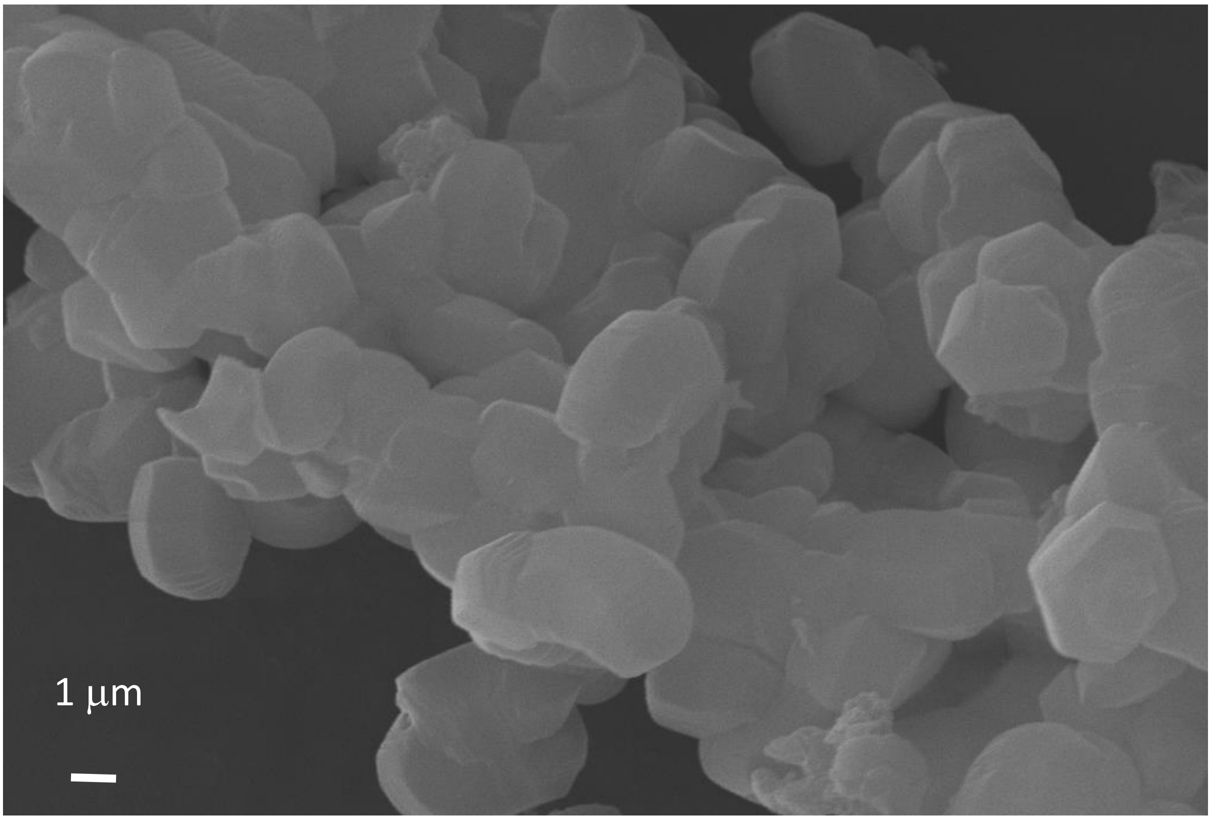

SM4

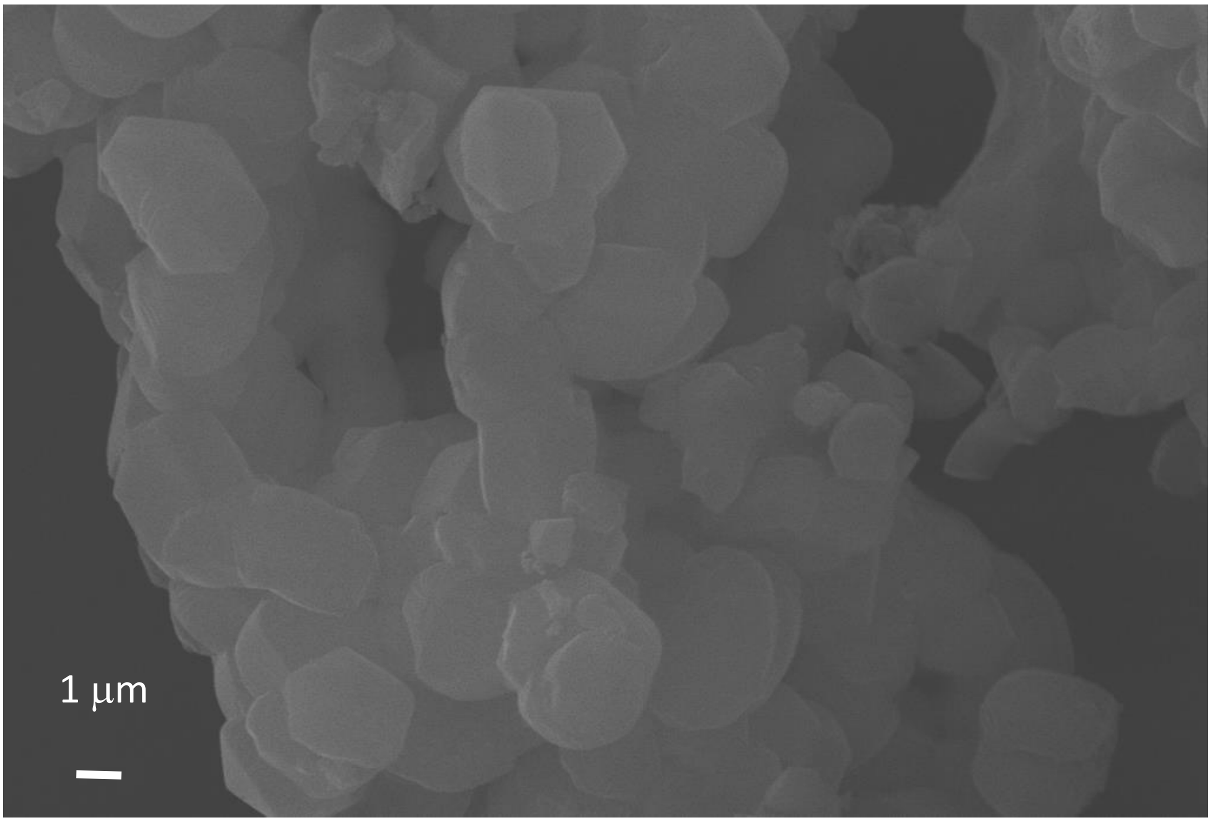

SM5

Figure S3e Scanning electron micrographs of the Syloid and SBA-15 carriers and metronidazole immobilized on their surface.

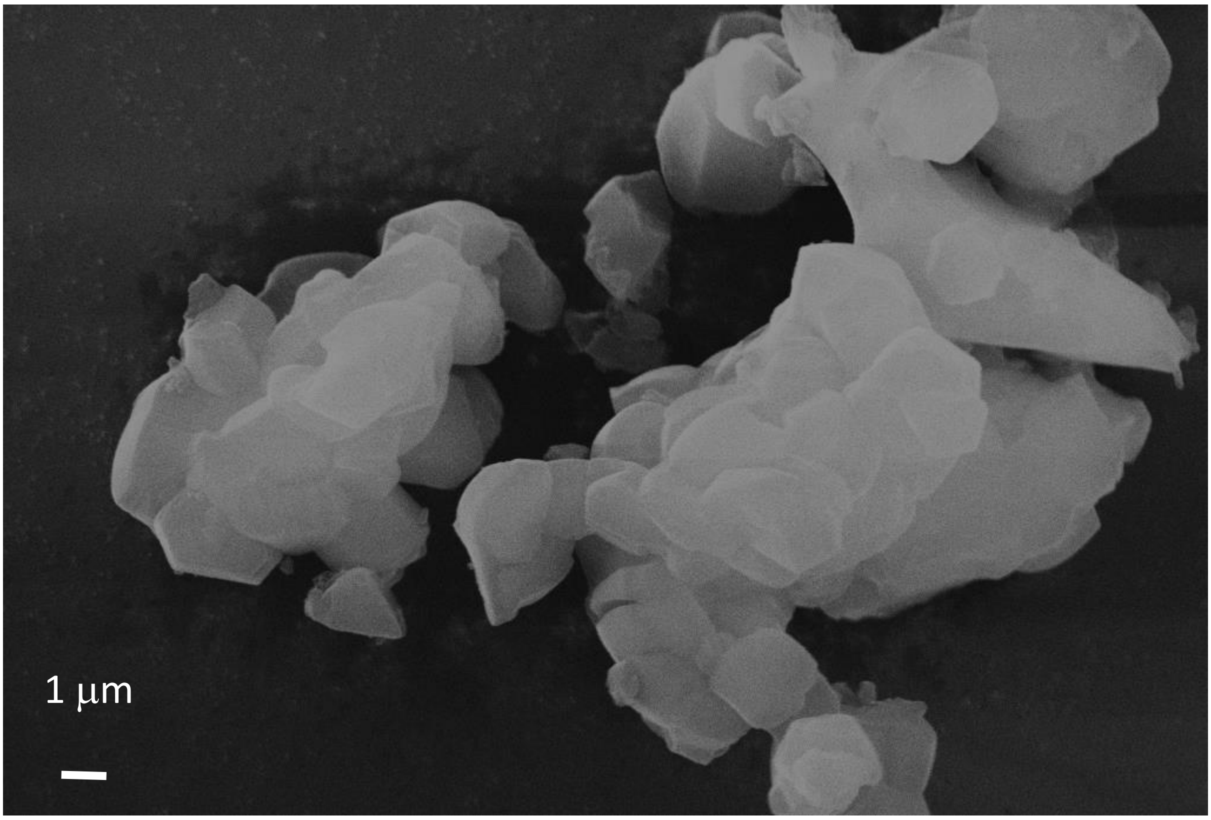

SM6

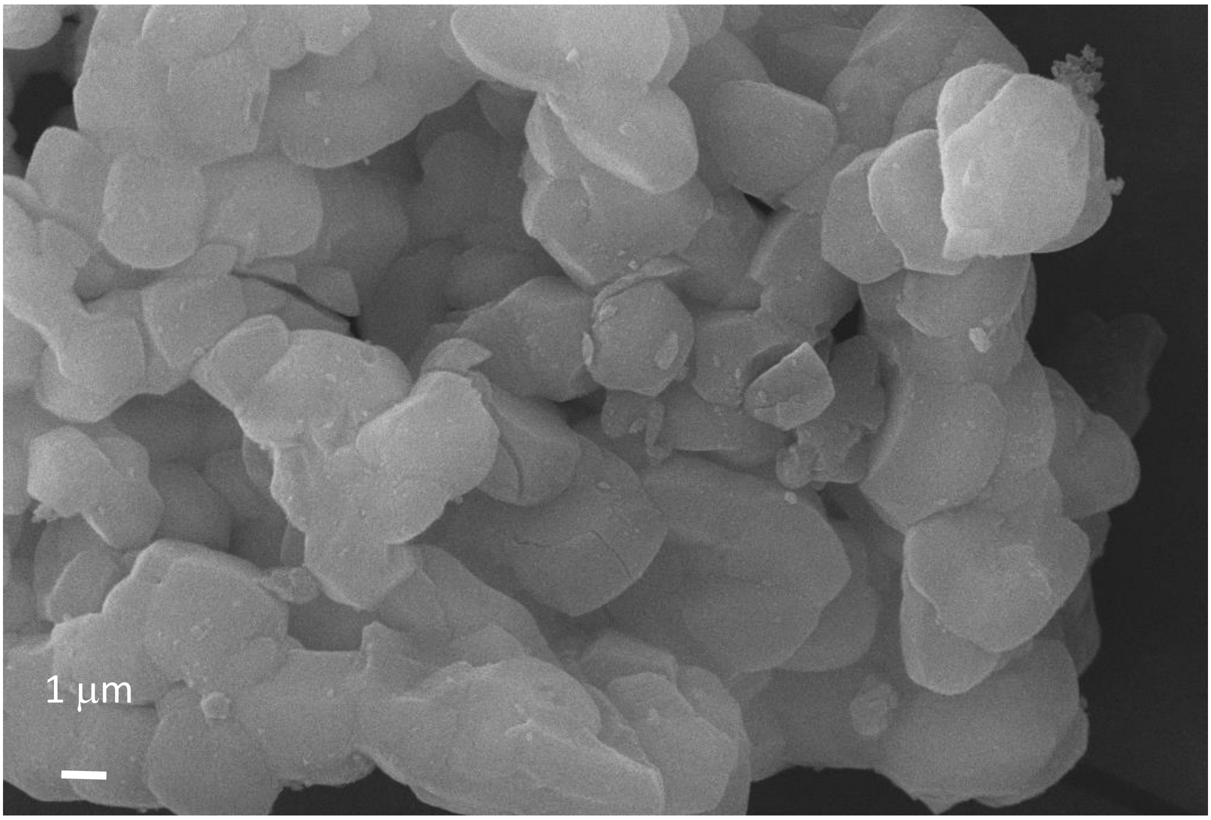

SM7

Figure S3f Scanning electron micrographs of the Syloid and SBA-15 carriers and metronidazole immobilized on their surface.

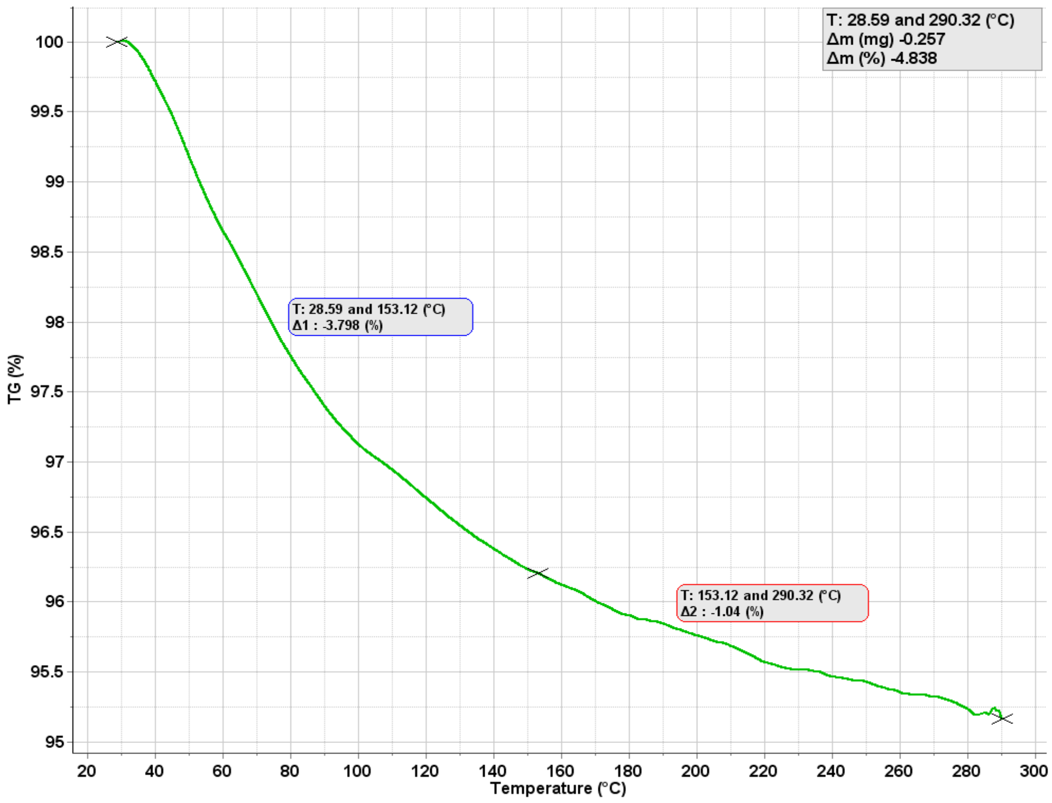

Syloid

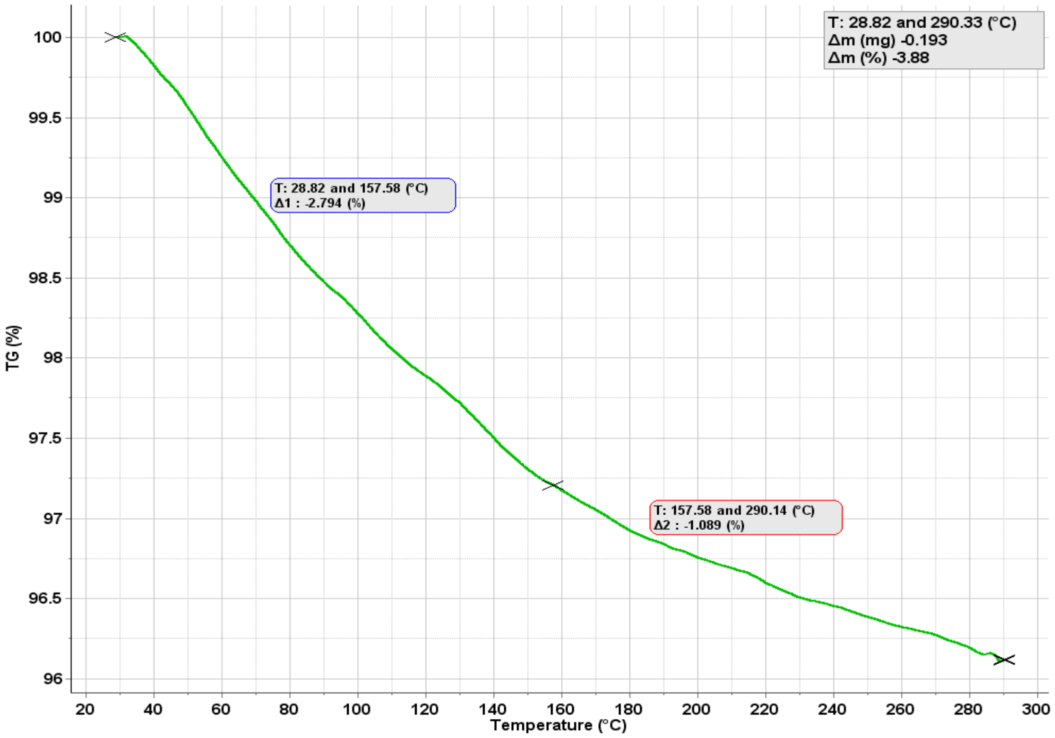

SyM1

Figure S4a Quantitative thermogravimetry of Syloid samples.

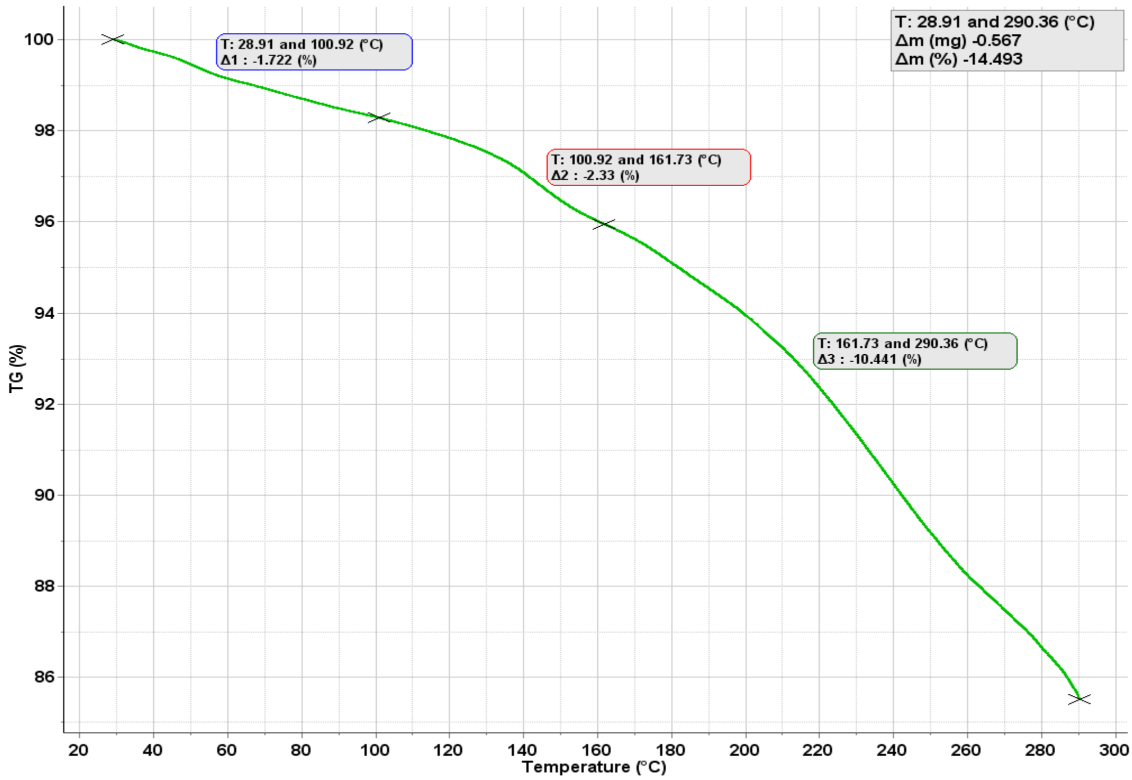

SyM2

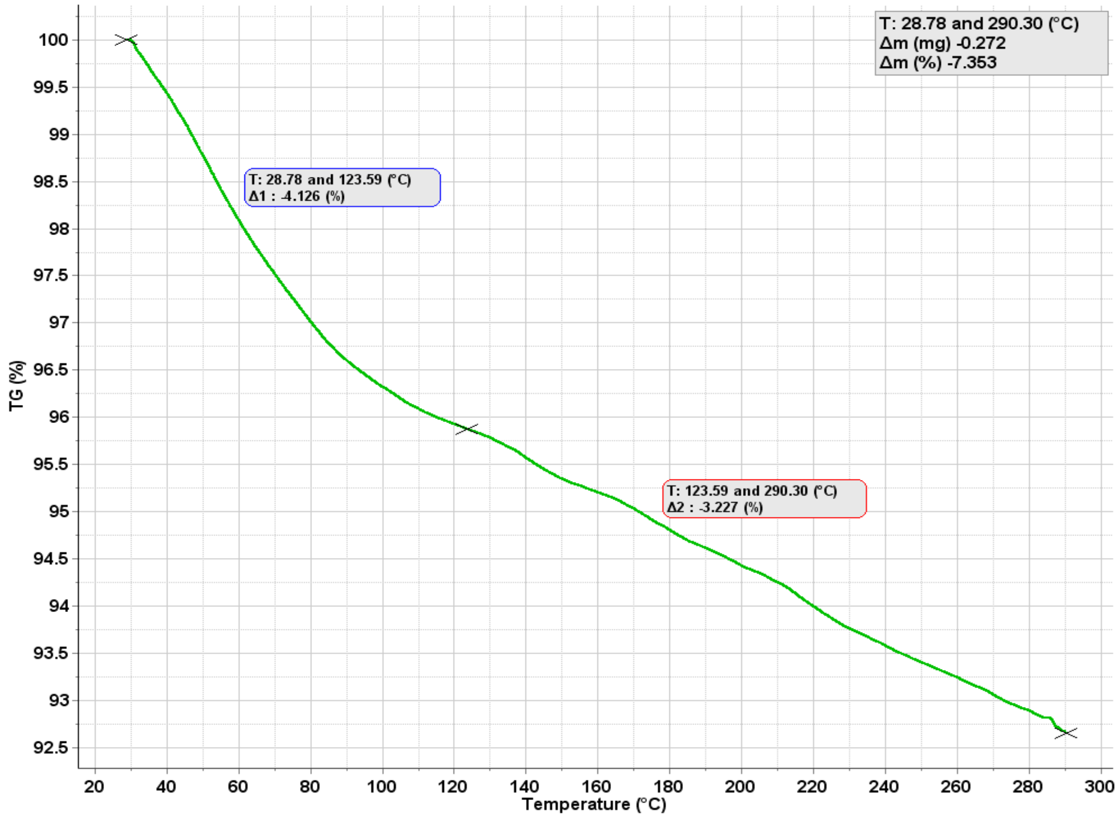

SyM3

Figure S4b Quantitative thermogravimetry of Syloid samples.

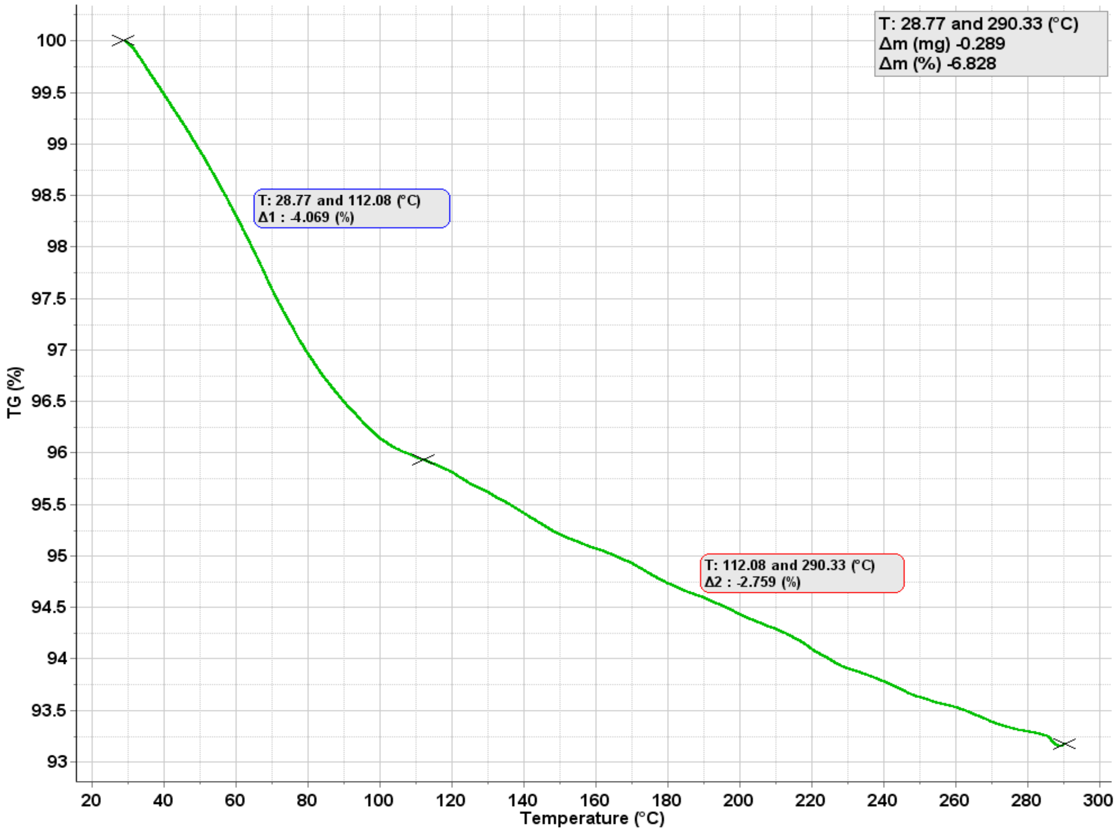

SM1

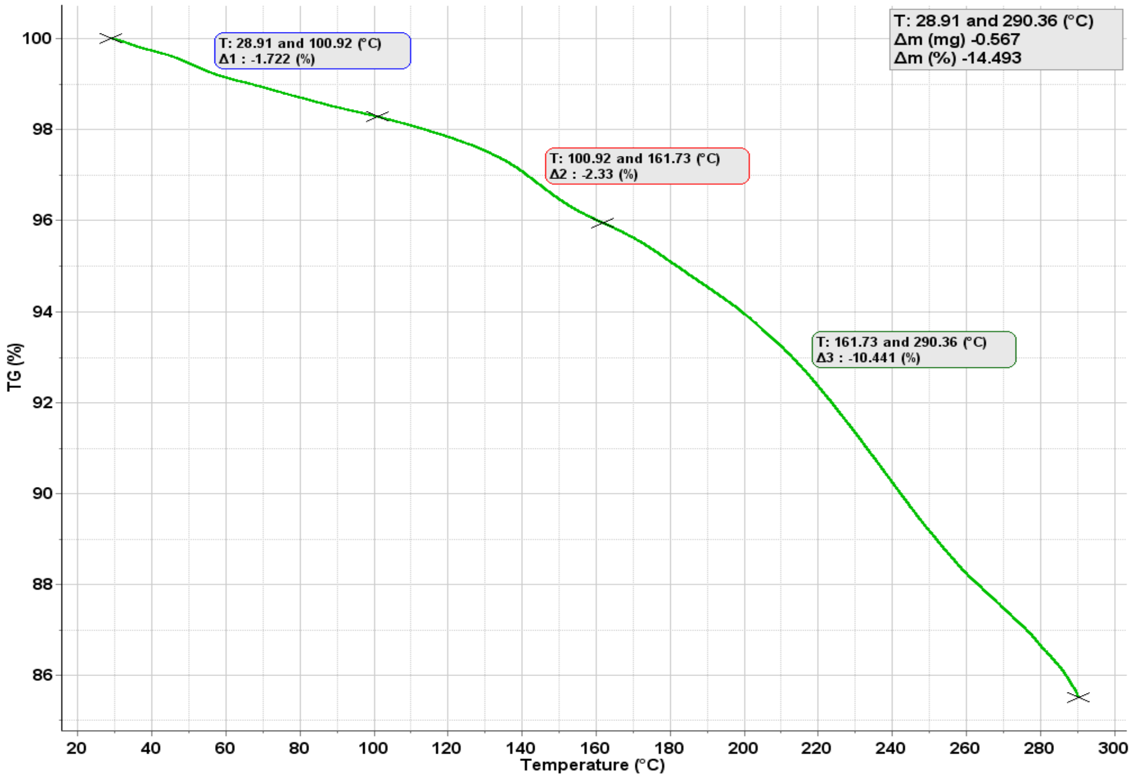

SM2

Figure S4c Quantitative thermogravimetry of SBA-15 samples.

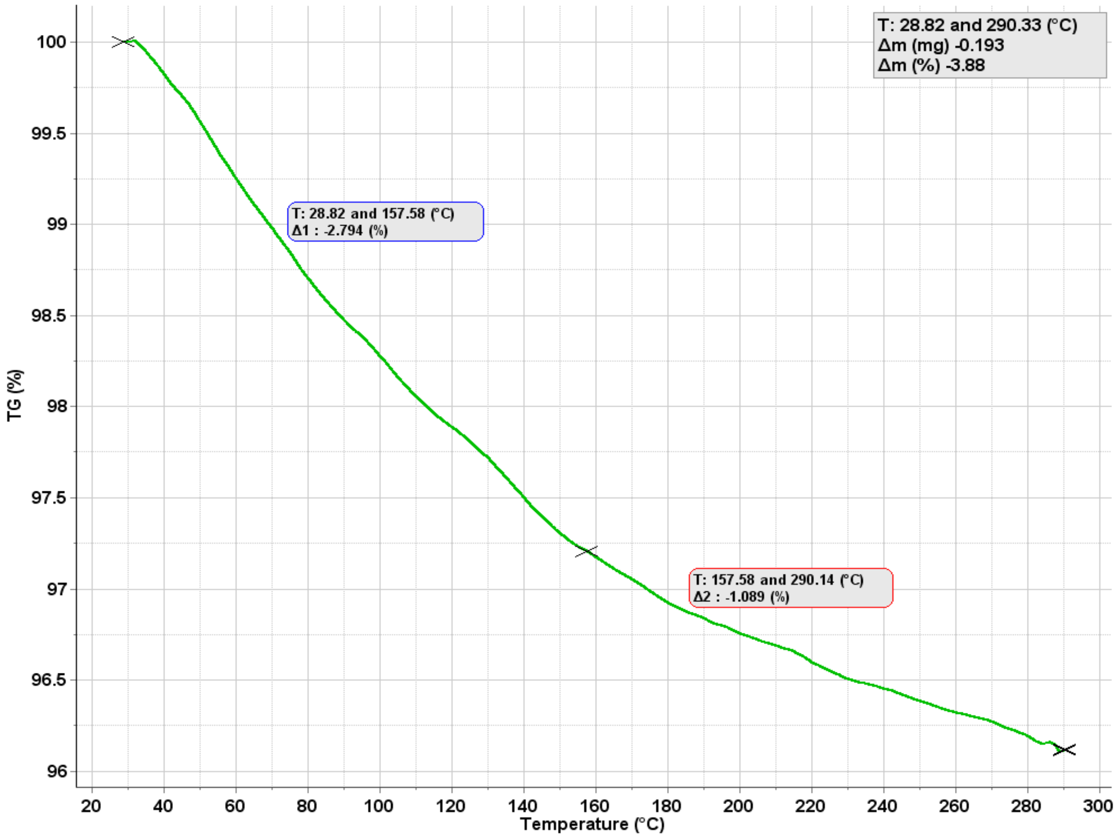

SM3

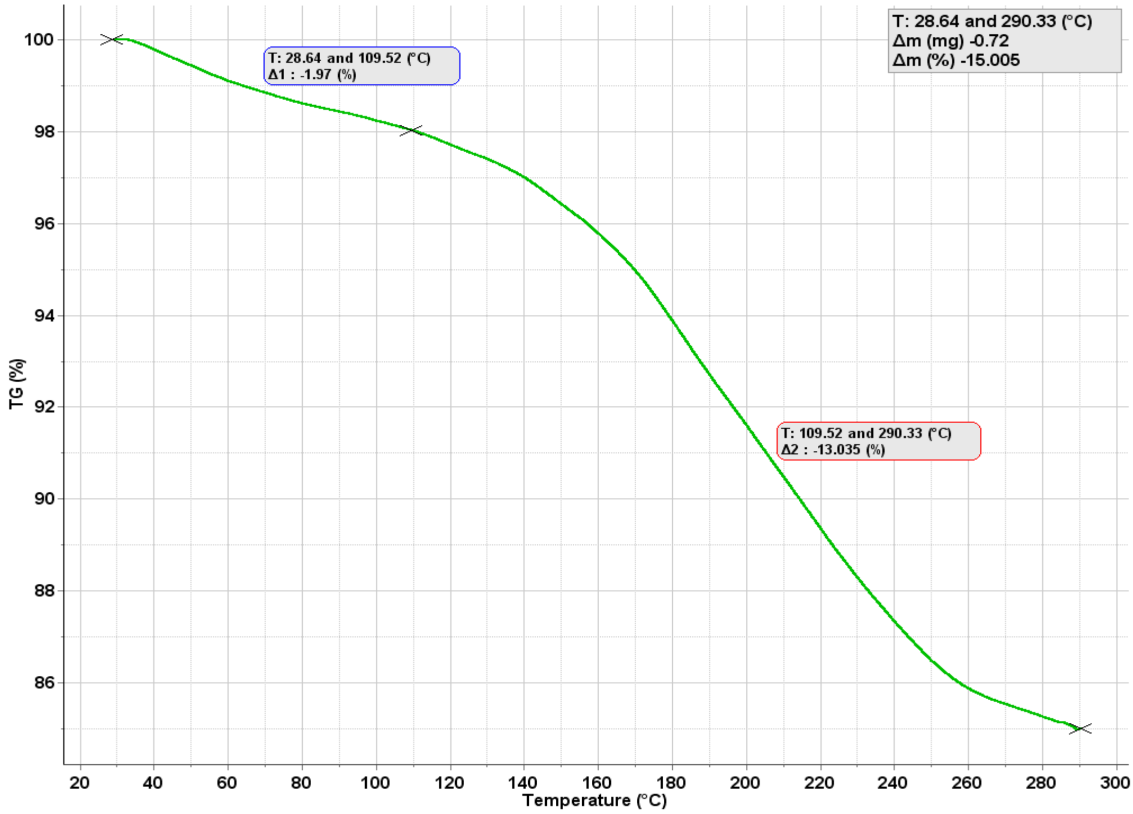

SM4

Figure S4d Quantitative thermogravimetry of SBA-15 samples.

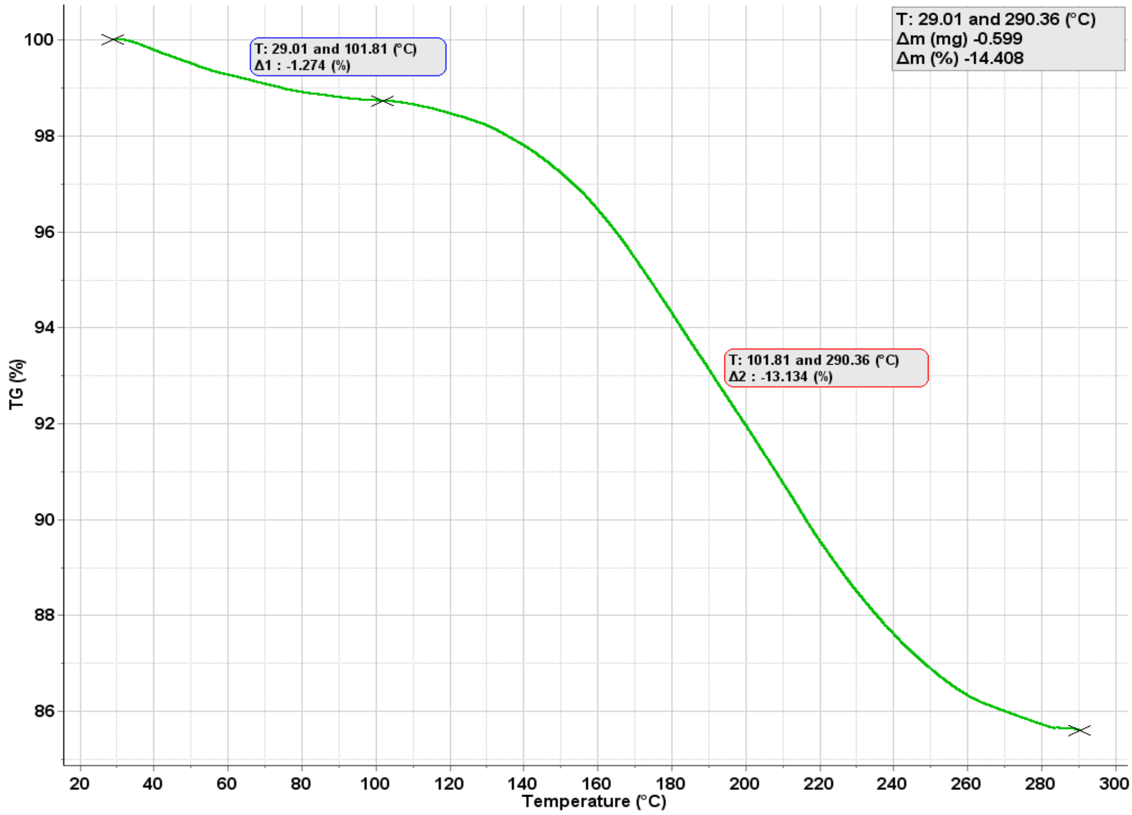

SM5

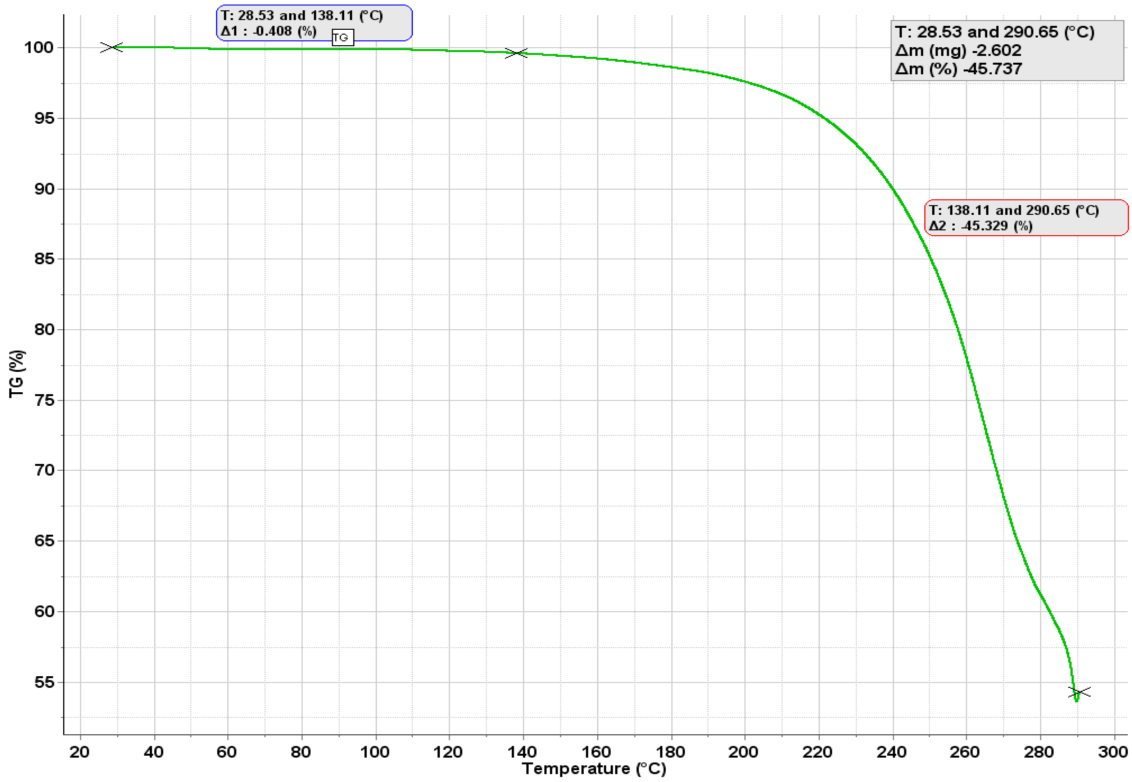

SM6

Figure S4e Quantitative thermogravimetry of SBA-15 samples.

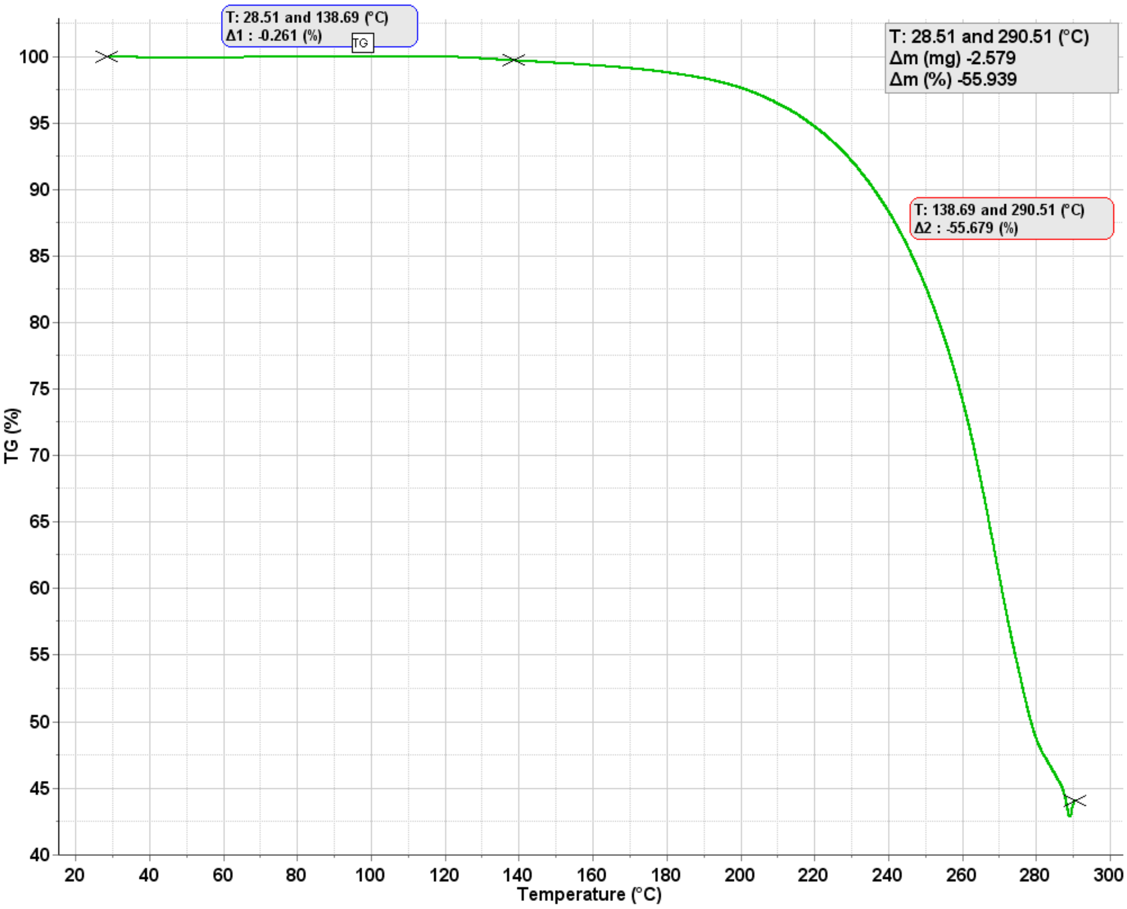

SM7

Figure S4f Quantitative thermogravimetry of SBA-15 samples.

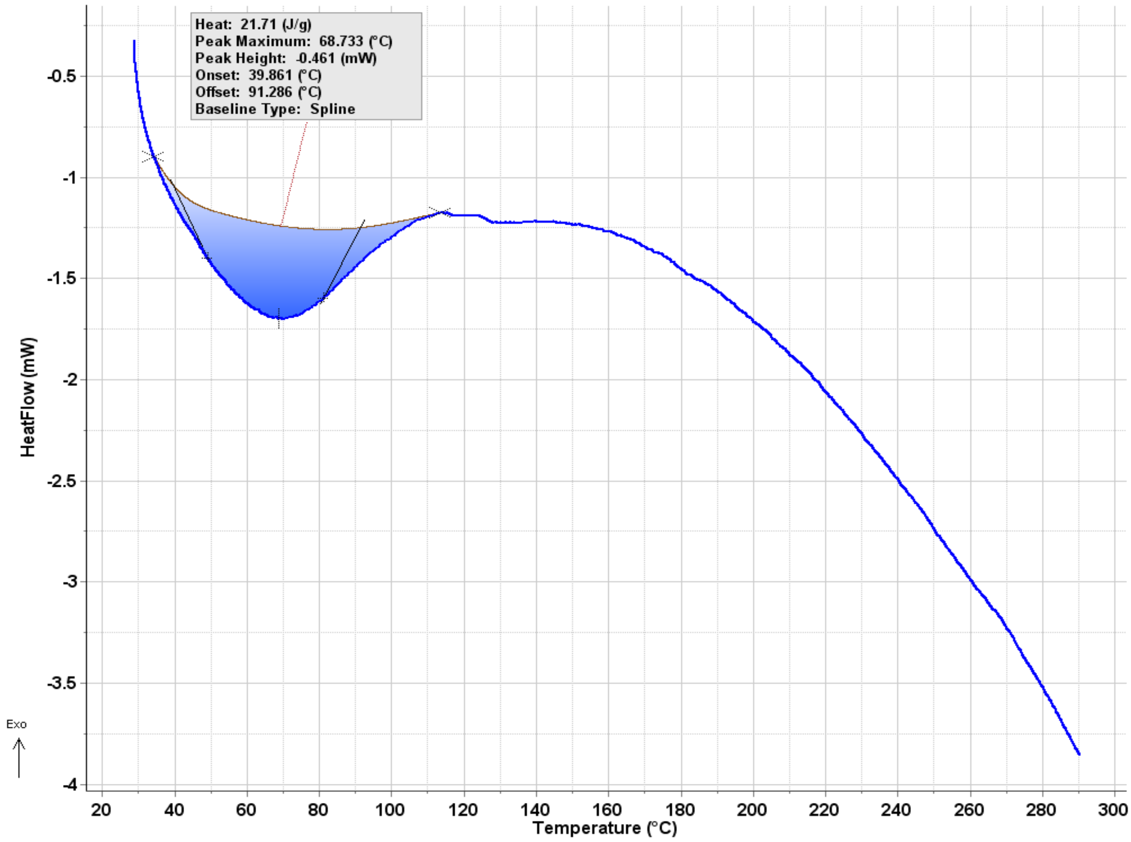

Syloid

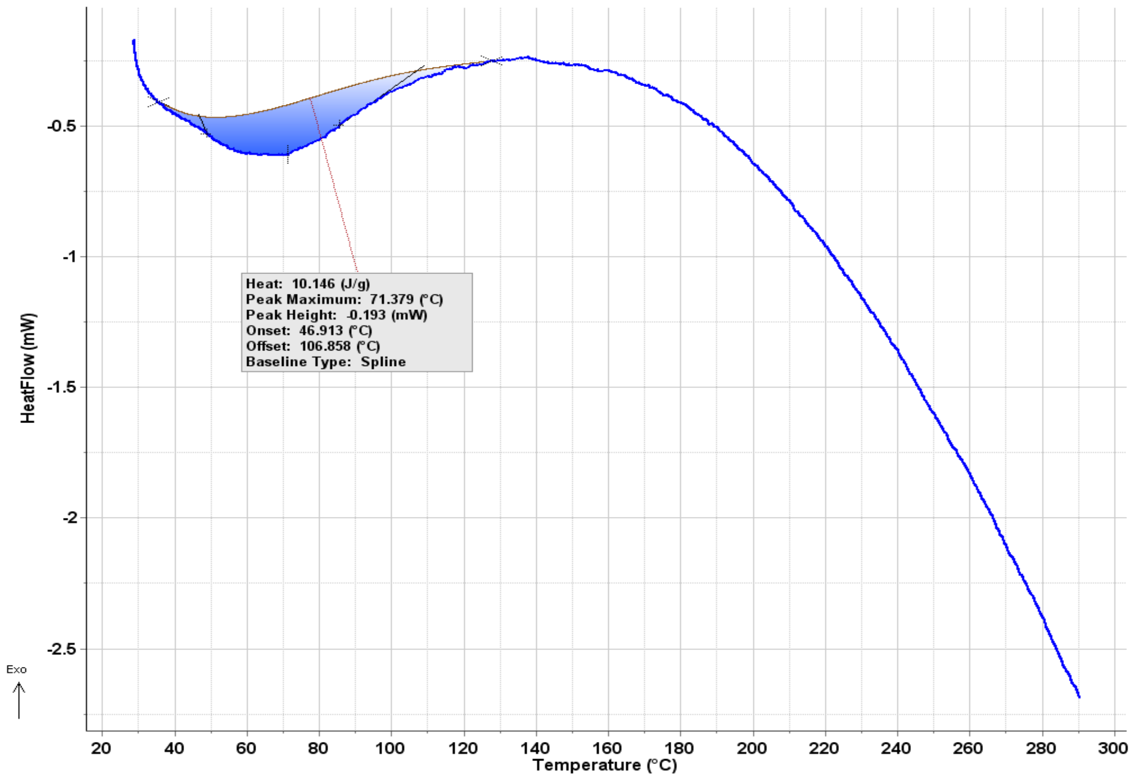

SyM1

Figure S5a Quantitative DSC of Syloid samples.

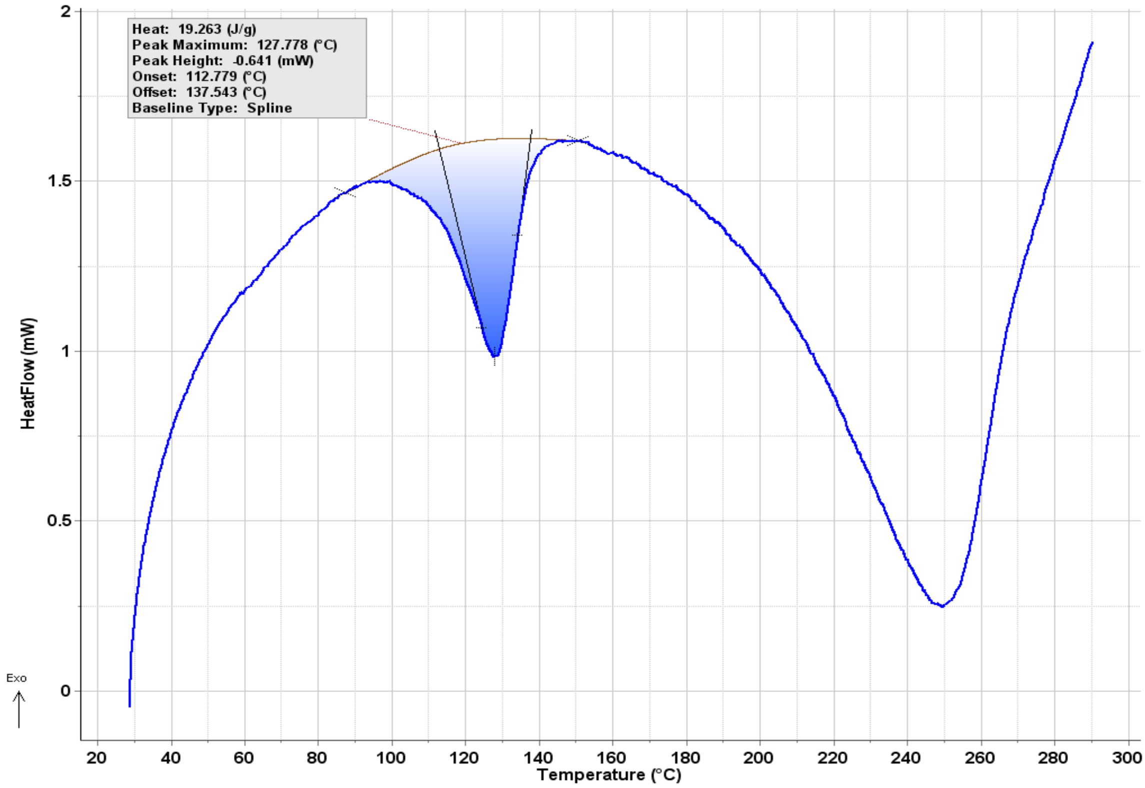

SyM2

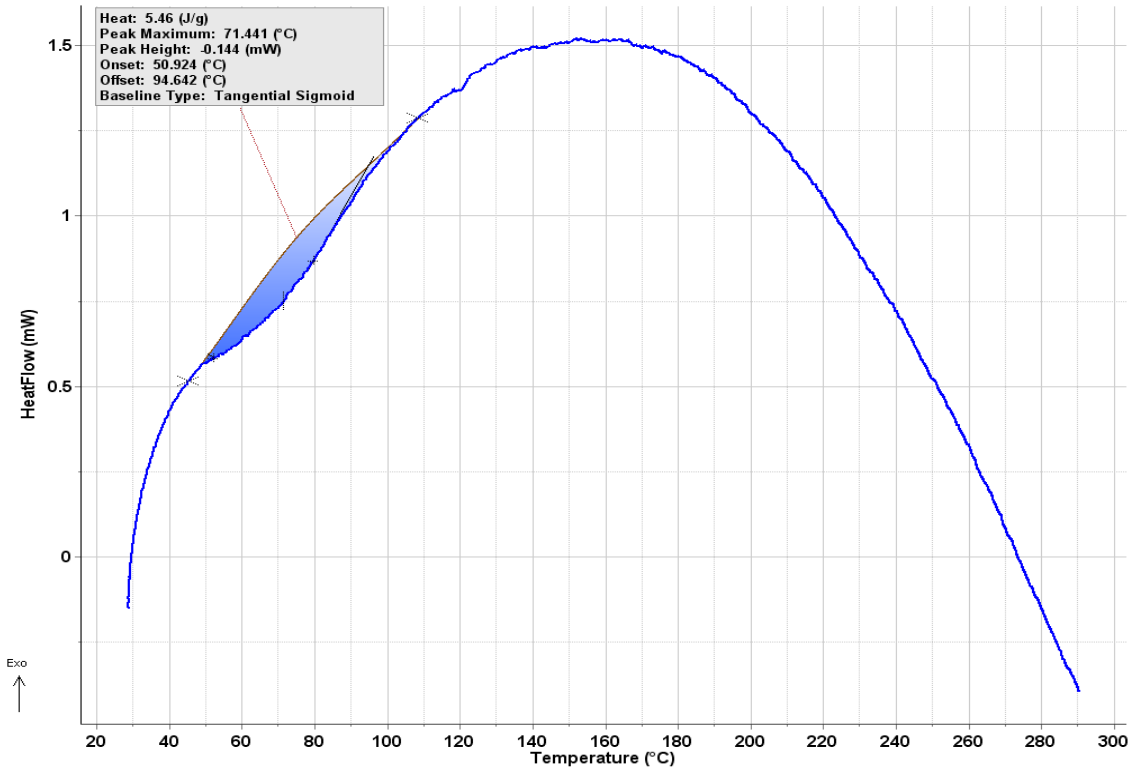

SyM3

Figure S5b Quantitative DSC of Syloid samples.

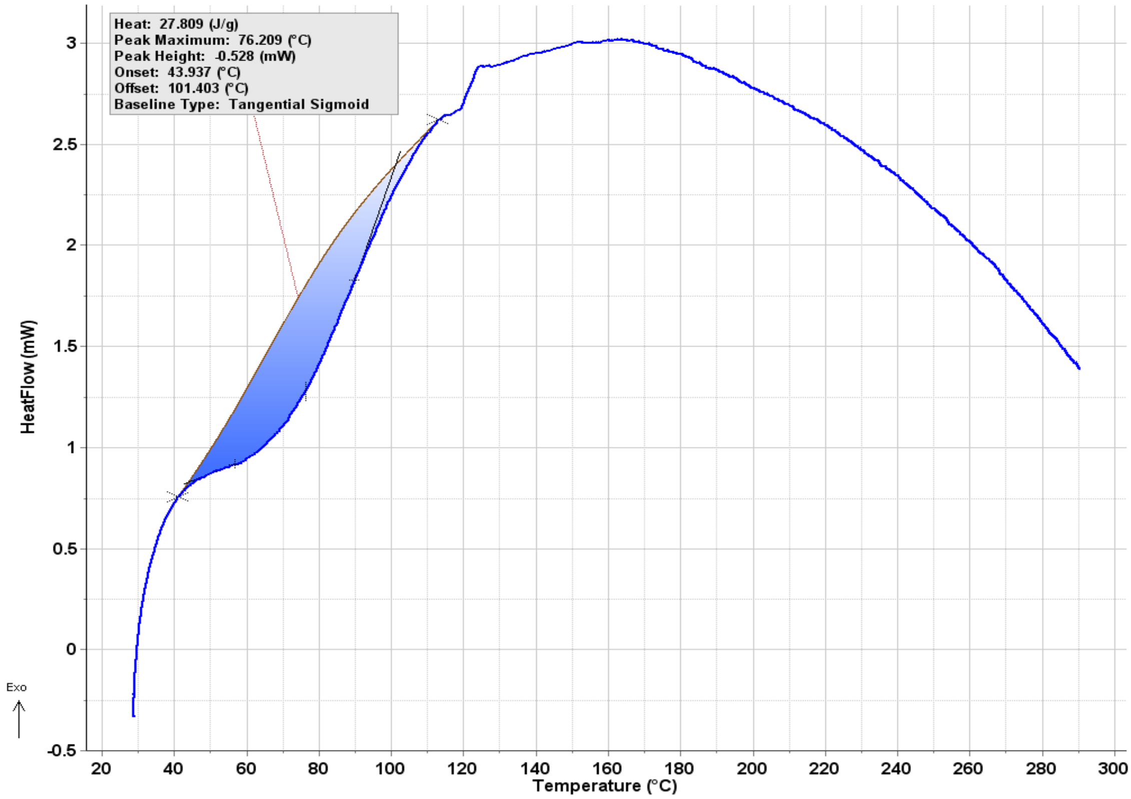

SM1

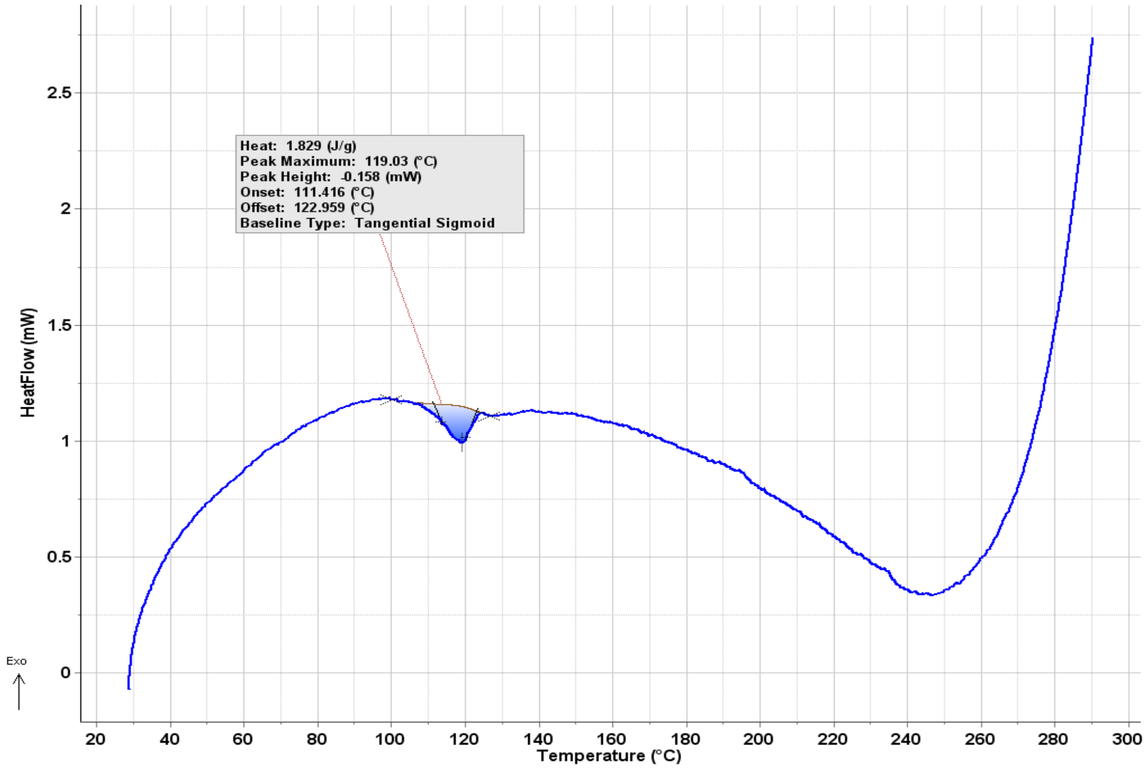

SM2

Figure S5c. Quantitative DSC of SBA-15 samples.

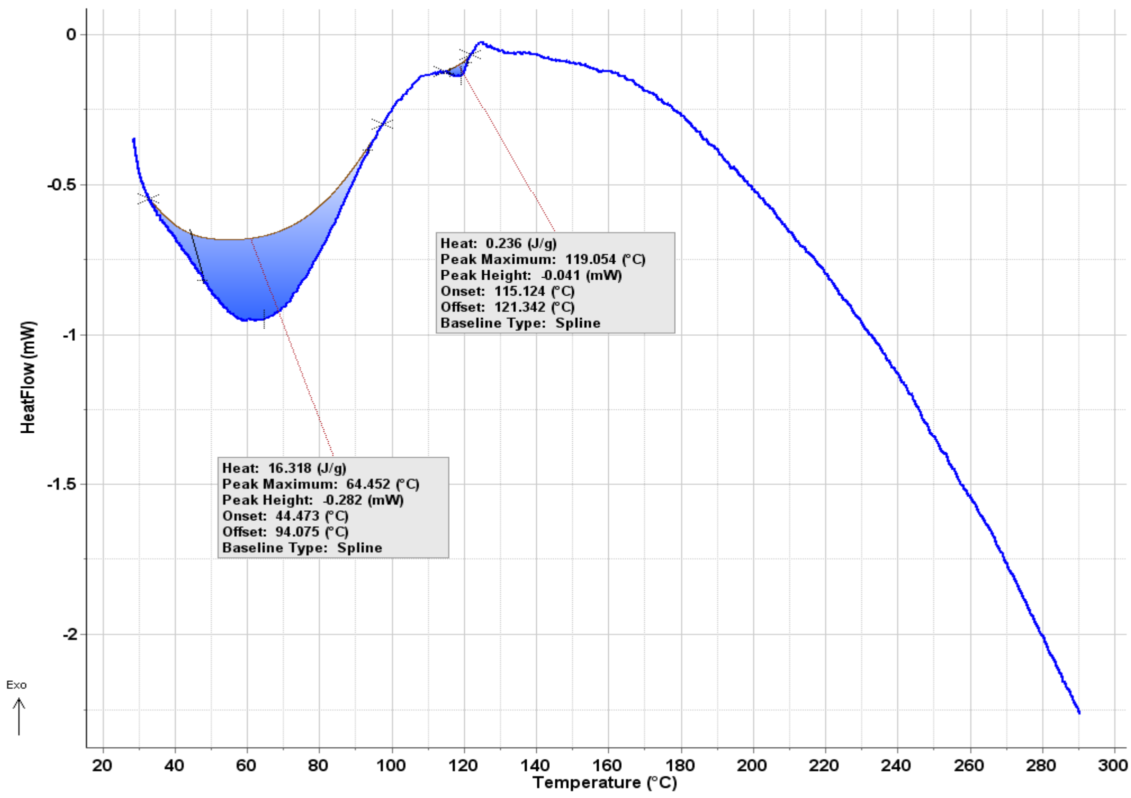

SM3

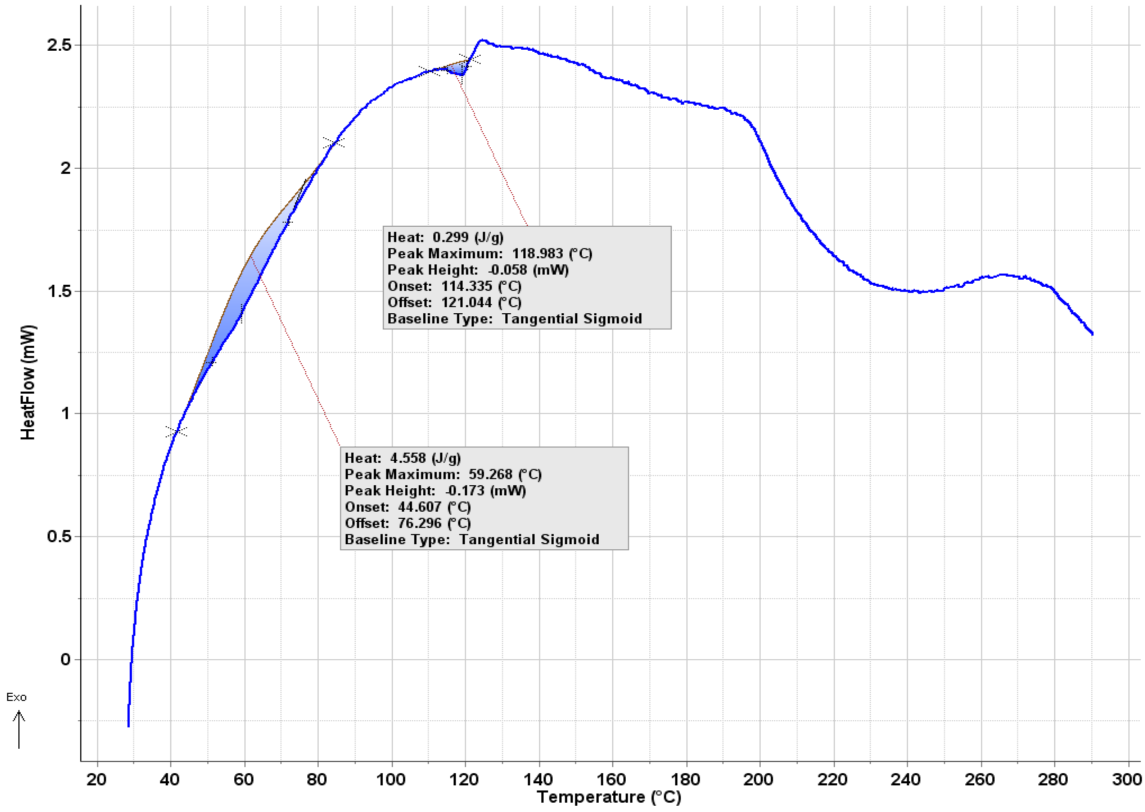

SM4

Figure S5d Quantitative DSC of SBA-15 samples.

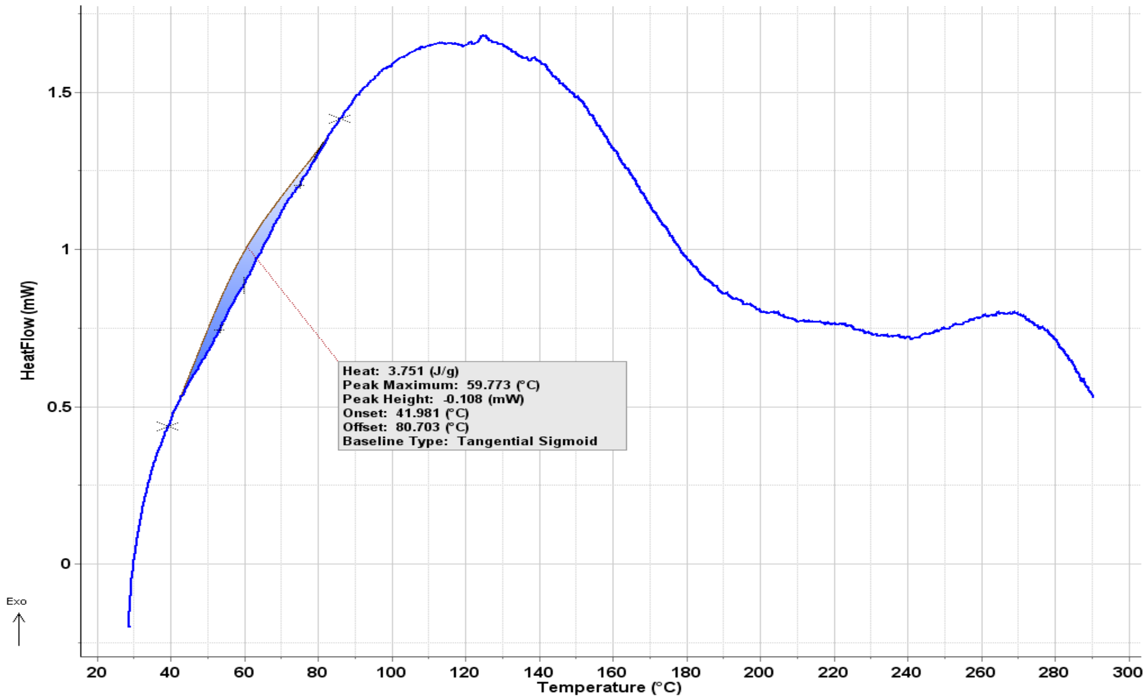

SM5

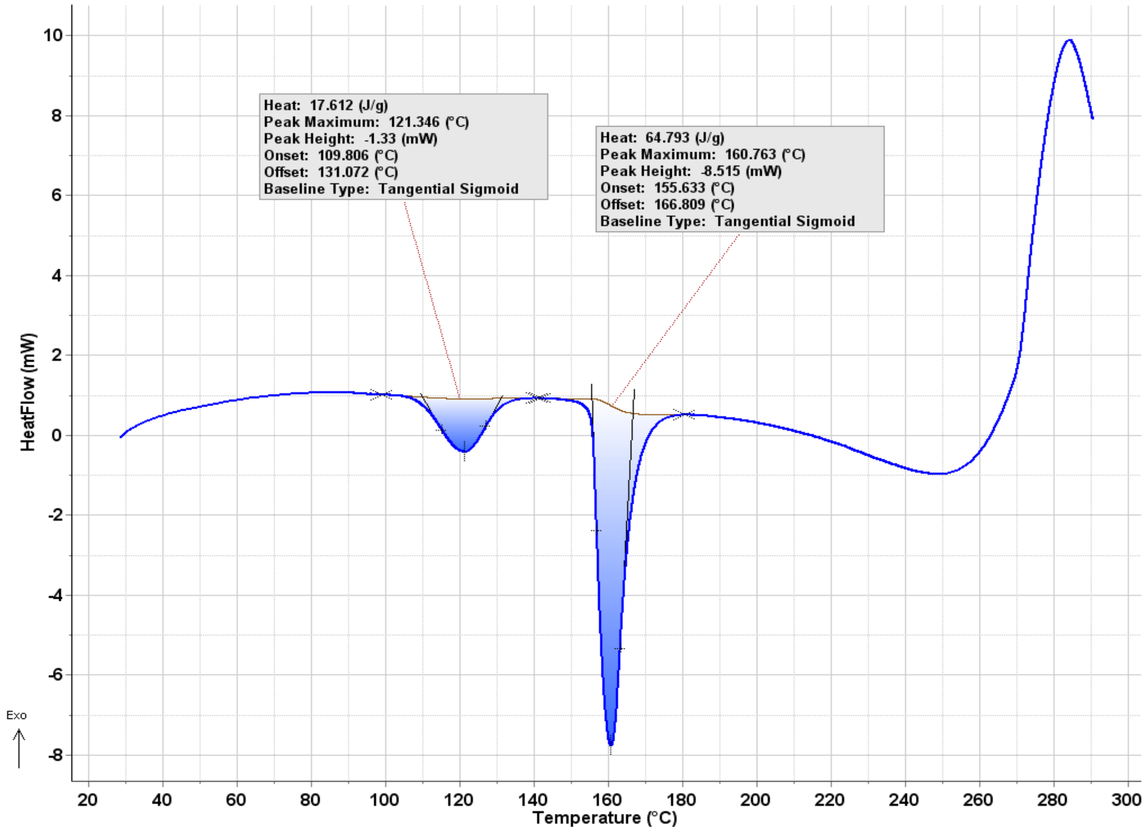

SM6

Figure S5e Quantitative DSC of SBA-15 samples.

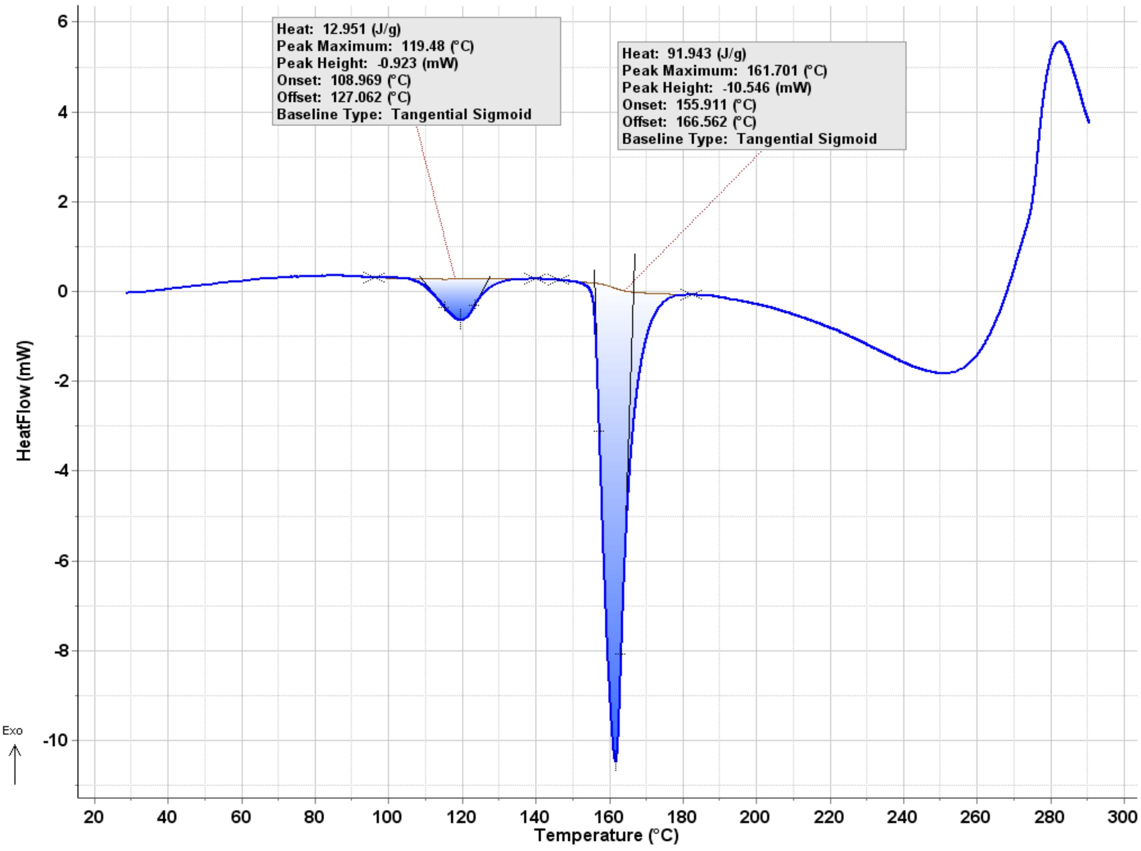

SM7

Figure S5f Quantitative DSC of SBA-15 samples.

Table S1. Differences of metronidazole percentage dissolution (%) as function of time.

Sample	from 0 to 5 min	from 5 to 10 min	from 10 to 15 min	from 15 to 30 min	from 30 to 60 min	from 60 to 90 min
MTZ	59.7	14.6	2.8	2.9	0.9	1
SyM2	54.8	2.5	5.8	11.4	2.2	10.1
SM2	58.5	1.1	4.5	8	10.2	7.4
SM6	58.7	2.5	17	8.3	2.5	1
SM7	59.4	4.8	8.2	8.6	10.2	4.9

Table S2. Dissolution speed (% *min*$^{-1}$) of metronidazole and its release speed from the composites as function of time.

Sample	from 0 to 5 min	from 5 to 10 min	from 10 to 15 min	from 15 to 30 min	from 30 to 60 min	from 60 to 90 min
MTZ	11.9	2.9	0.56	0.19	0.03	0.03
SyM2	10.9	0.5	1.16	0.76	0.07	0.34
SM2	11.7	0.2	0.90	0.53	0.34	0.25
SM6	11.7	0.5	3.40	0.55	0.08	0.03
SM7	11.9	0.96	1.64	0.57	0.34	0.16
